# Inelastic Responses and Finite Element Predictions of Fiber Cementitious Composite and Concrete Columns

**DOI:** 10.3390/ma14092180

**Published:** 2021-04-24

**Authors:** Chang-Geun Cho, Sun-Ju Lee

**Affiliations:** Department of Architectural Engineering, Chosun University, Gwangju 61452, Korea; ssdj900@naver.com

**Keywords:** flexibility formulation, SHCC, fiber cementitious composites, concrete column

## Abstract

In this research, reinforced concrete (RC) and strain-hardening cementitious composite (SHCC) columns subjected to lateral loads combined with a constant load were investigated, both by experiments and predictions, with two distributed inelastic finite element models established by the stiffness and flexibility formulations. SHCC applied in the column plastic hinge region could not only enhance the lateral load and displacement capacities of columns but also offer effective advantages in the control of bending and shear cracks induced by multiple microcracks, the prevention of the spalling of cover concrete, and the resistance to buckling of steel bars. With the layered cross-sectional approach using constitutive laws of SHCC considering a proposed model of the post-cracked high-ductile tensile characteristics, as well as concrete and reinforcing steel bars, an inelastic beam-column finite element model was presented with a distributed flexibility formulation. In comparison with experiments concerning the RC and reinforced strain-hardening cementitious composite (R-SHCC) columns, the current flexibility method showed relatively accurate estimations in the lateral load and displacement responses of column systems as well as in localized nonlinear responses of cross-section as estimated in axial strains of longitudinal reinforcing steel bars. In comparison with the stiffness method, the current flexibility method gave more accurate solutions at both element and structural levels, as manifested in the experiments and analysis solutions.

## 1. Introduction

Sometimes reinforced concrete (RC) columns at the lower stories of a building, under medium or strong earthquake loads, can experience severe damages caused by the progress of bending and shear cracks, the spalling of cover concrete, and the yielding and buckling of longitudinal reinforcing steel bars, and the damage to the the column, especially, were localized to concentrate in the column plastic hinge region [1,2,3].

The nonlinear solution of RC beam-column finite element (FE) model is important in applications of performance-based evaluation, design, and retrofit of concrete structures in medium or strong earthquake zones. The inelastic analyses of RC frames were initially concentrated at the ends of beams and columns by means of nonlinear springs located at the member ends [4,5]. An improved formulation ofon the inelastic behavior of RC structural members was possible with distributed nonlinearity models with classical plasticity theory or explicitly derived by discretization of the cross section into fibers [6,7,8]. The distributed nonlinearity frame model were formulated with the displacement-based finite element method using cubic Hermitian polynomials to approximate the deformations along the element. However, the shape functions of a beam element in the inelastic state were assumed as same as that in the elastic state, and the well-known stiffness formulation could not satisfy the equilibrium assumptions in an element.

Concrete or cementitious binders mixed with steel, metal, or synthetic fibers could be innovated as their mechanical characteristic from a brittle material weakened by tensile cracks to a post-cracked high ductile material induced by multiple microcracks. Many researchers, for this purpose, have developed several types of fiber cementitious composites, such as engineered cementitious composites (ECC), high-performance fiber-reinforced cementitious composites (HPFRCC), and strain-hardening cementitious composites (SHCC) [9,10,11,12,13,14]. Considering the weakness of concrete in post-cracked tensile performances, fiber-reinforced cementitious composites, ECC, or SHCC could innovate the load and deformation capacities as well as the bond and shrinkage performances of concrete structural members under flexure [14,15,16,17,18]. In the design of RC structural walls, column members, beam-column joints, reinforcing steel bars could be minimized without loss of strength and ductility performances by applying fiber cementitious composites [3,10,11].

In the current study, SHCC was applied as an attempt to improve structural performances of conventional RC structural columns and the applications were evaluated by a series experiments and finite element predictions on cantilever columns subjected to lateral loads combined with a constant load. As for the novelties of this research: first, the efficiency of SHCC instead of concrete at column plastic hinge regions was evaluated with cases of tied and non-tied steel bars. Second, in order to ensure more accurate solutions both in global load and displacement responses and in local damages and local nonlinear responses at critical column plastic hinge regions, an inelastic flexibility beam-column finite element analysis model was newly developed for the analysis of R-SHCC applied concrete composite columns. Moreover, the cross-sectional force and deformation relation was newly established with constitutive laws of SHCC as a proposed model of the post-cracked high-ductile tensile characteristic, and the sectional nonlinear responses were solved with integrations of the layered approach. Therefore, the element formulation was cast in a distributed inelastic beam-column finite element method in which the element state was determined by the convergence of a nonlinear solution in an element in order to satisfy equilibrium within the element. The following sections of this article consist of the manufacture and mechanical characteristics of SHCC, the formulation and modeling of the flexibility beam-column finite element for R-SHCC and concrete composite columns, manufactures and loading setups of column specimens, experiments and finite element predictions of columns, and finally conclusions are presented.

## 2. Properties of SHCC and Manufactures of Column Specimens

### 2.1. Mixing and Properties of SHCC

**Figure 1:**
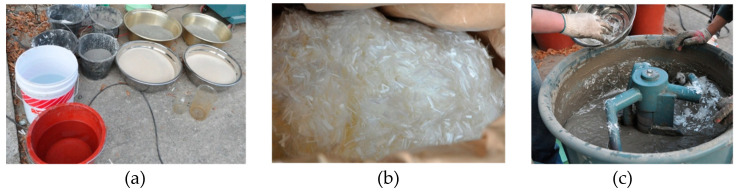
Mixing and manufacturing of SHCC, (**a**) mixing materials, (**b**) PVA fibers, (**c**) mixing of SHCC.

In order to improve tensile brittle characteristics of concrete, as shown in Figure 1, fiber-reinforced SHCC was manufactured by mixing of Polyvinyl alcohol (PVA) short fibers made by Kuraray Co., LTD, Osaka, Japan, ordinary Portland cement (OPC) made by Halla Cement Co., LTD, Seoul, Korea, fine aggregates of maximum grain size 0.25 mm, water, a high-range water-reducing admixture, and several admixtures, at a certain ratio as presented in past research [10,11]. The mixing of PVA short fibers in cementitious binders could renovate the tensile brittleness of cementitious binders so as to give high-ductile tensile behaviors after cracks have taken place. The PVA fibers used here had a short length of 12 mm and a tensile strength of 1600 MPa with a surface treated by an oiling agent [10,11], and it was well known from several researches that a mixture of content of PVA fibers in fiber cementitious composites was suitable for cases of a fiber volume fraction of 1.5~2.0% [11,17,18]. The SHCC manufactured in the current research mixed with minimum use of PVA fibers as a volume fraction of 1.5% to reserve high fluidity for placing of fresh SHCC between narrow spaces of reinforcing steel bars, such as in column plastic hinge regions. The slump flow from the slump cone test on mixed fresh SHCC was measured as an average of 668 mm.

**Figure 2:**
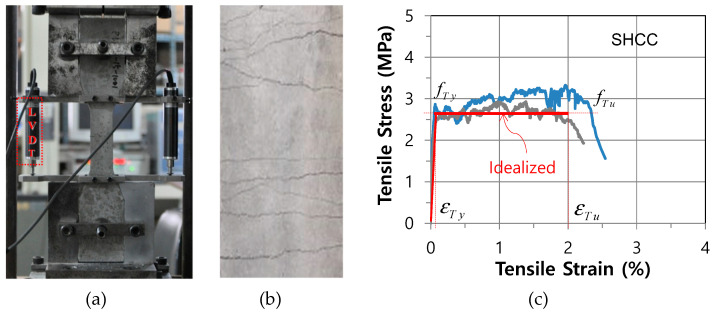
Direct tensile test: (**a**) test setup; (**b**) microcracks; (**c**) post-cracked tensile behavior and model.

For the compressive test on hardened SHCC, three specimens of SHCC from cubic molds were manufactured and the compressive strength of SHCC at 28 days was measured as an average of 39.2 MPa. To evaluate the tensile characteristic of hardened SHCC, on the other hand, a direct uniaxial tensile test was carried out using a 10 kN capacity universal testing machine (UTM) by controlling the displacement of 0.2 m/min, as shown in Figure 2.

The specimens had a dimension with a 30 mm × 30 mm cross-section and a length of 330 mm [3]. After curing in water for 28 days, from direct tensile tests, the specimens finally failed after multiple microcracks, as shown in Figure 2b, and the tensile stress and strain responses of the hardened SHCC specimens were also obtained, as shown in Figure 2c. After the initial cracks had taken place on the surface, the SHCC specimen showed not a brittle but a high-ductile characteristic as the tensile stress was sustained until the limit tensile strain reached up to about 2.0%. The high-ductile tensile characteristic was caused by multiple microcracks which were controlled by short fibers within cementitious binders [9,10,11,18].

### 2.2. Manufacture of RC and R-SHCC Column Specimens

**Figure 3:**
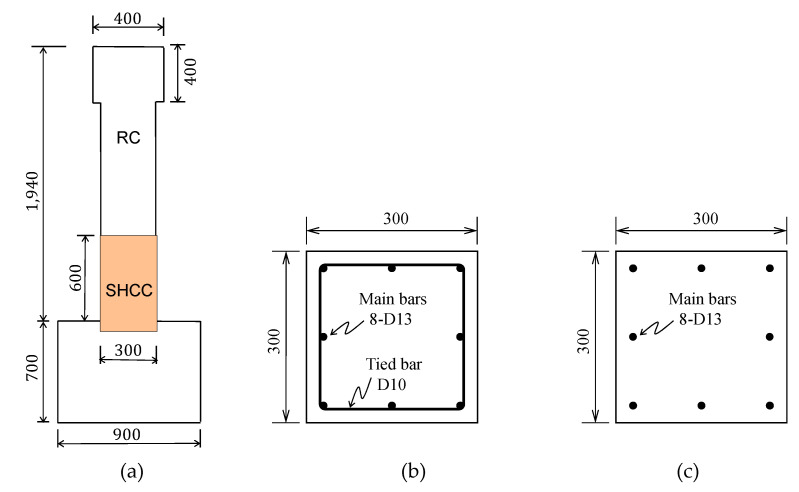
Layout of column specimen with cross-section (unit: mm): (**a**) dimensions; (**b**) cross-section (tied bars); (**c**) cross-section (no Tied bars).

Three column specimens were manufactured with representing the first-storey column between the footing and the inflection point. As shown in Figure 3, each column specimen was fixed to the column base upon a reinforced concrete footing as a cantilever column and had a cross-section of 300 mm × 300 mm. The height of column was 1940 mm with the height of 400 mm in the head part of column.

**Figure 4:**
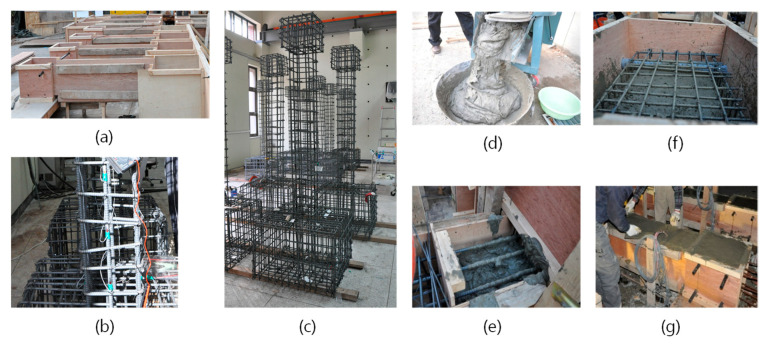
Manufacturing process of column specimens: (**a**) Wood formwork; (**b**) Steel gauges; (**c**) Reinforcements; (**d**) Fresh SHCC; (**e**) Placing SHCC; (**f**) Placing concrete; (**g**) Finishing.

Three column specimens were presented with design variables as shown in Table 1. One specimen, RC-0, was a conventional RC column and two specimens, RSH-s and RSH-n, were specimens of RC and R-SHCC composite columns in which the lower part of the column has the length of 600 mm from the column base, placed not in concrete but SHCC in order to compare local damages and performances of the column plastic hinge region. For a specimen of RSH-n, the part of SHCC in the column plastic hinge region had no reinforcing steel tied bars. The practical manufacturing process of specimens is shown in Figure 4.

Reinforcing steel bars produced in Korea had the yielding stresses for the main steel bar, D13, and the tied steel bar, D10, as 385 MPa and 383 MPa, respectively. Concrete was mixed with ordinary Portland cement, crushed stones with a maximum aggregate size of 20 mm, sand and admixtures, and the uniaxial compressive strength of hardened concrete in cylinder test was recorded as the average of 29 MPa after curing at 28 days.

**Figure 5:**
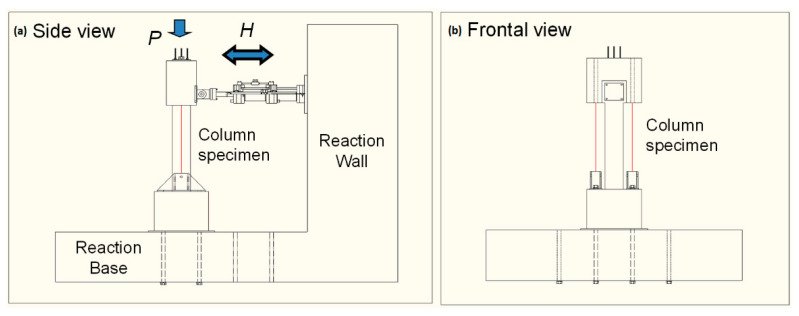
Schematic of column loading test: (**a**) Side view of specimen setup; (**b**) Frontal view of specimen setup.

Each column specimen was installed on the reaction base and the reaction wall as illustrated in Figure 5. The lateral column load H was applied through a reaction wall equipped with a 100 kN capacity actuator according to a displacement controlled loading [3]. External steel tendons were attached between the pin and the loading frame in order to apply the axial load of the column P as 190 kN during the lateral loading stage. The specimens were equipped with a displacement transducer at the top of the column to measure and control the lateral displacement of the column. The strain gauges were attached, from the column base to the height of 600 mm, to the longitudinal reinforcing steel bars at intervals corresponding with the spacing of tied steel bars.

## 3. Beam-Column Finite Elements with Flexibility Formulation

In order to predict inelastic responses of RC and R-SHCC columns, an inelastic distributed frame finite element model was applied using an isoparametric finite element formulation combined with a layered cross-sectional approach.

### 3.1. Beam-Column Finite Element Formulation

**Figure 6:**
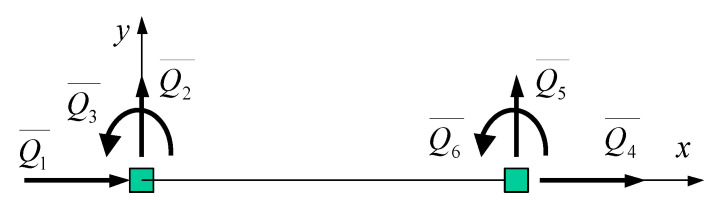
FE with rigid body modes.

The current R-SHCC column element was formulated using a flexibility beam-column finite element, which was basically an extended version of a RC beam-column element model originally presented by Spacone et al. [19]. In the current inelastic element formulation, the convergence of equilibrium within an element was adopted by two iterations in order to improve the accuracy of the solution of the nonlinear problem. For element formulations, a beam-column finite element was commonly formulated with rigid body modes as shown in Figure 6.

**Figure 7:**
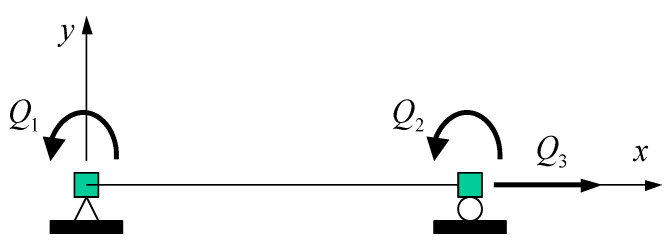
FE without rigid body modes.

The element formulation discussed here was established in the local coordinate system without rigid body modes, as shown in Figure 7; thus, the element nodal force vectors Q and corresponding element nodal deformation vectors q were measured with respect to the cord connecting the two end nodes.

Denoting with increments of the corresponding quantities, the section deformation D(x) and section force fields d(x) could be expressed, Equations (1) and (2):

(1) Δd(x) = B(x) Δq

(2) $$\Delta D\left(x\right)\;=\;B_{Q}\left(x\right)\;\Delta Q$$

where matrices B(x) and $B_{Q}\left(x\right)$ were defined as the deformation and force interpolation function matrices, respectively. The incremental section constitutive relation could be expressed as the form Equation (3):

(3) $$\Delta d\left(x\right)\;=\;f_{t}\left(x\right)\;\Delta D\left(x\right)\;\;+\;\;r(x)$$

where $f_{t}\left(x\right)$ and r(x) were the section tangential flexibility and section residual deformations, respectively. The section residual deformations could be known as the linear approximation to the deformation error made in the linearization of the section force-deformation relation.

The element relation between force increments and corresponding deformation increments could be obtained from the principle of virtual forces, δQ, Equation (4):

(4) $$\delta Q^{T}\;\Delta q\;=\;\int_{0}^{L}\;\delta D^{T}\left(x\right)\;\Delta d\left(x\right)\;dx$$

Upon rearrangement of above equations, the element flexibility equation could be derived as, Equation (5):

(5) $$\Delta q\;=\;F_{t}\;\Delta Q\;-\;s$$

where $F_{t}$ was the tangential element flexibility matrix derived as, Equation (6):

(6) $$F_{t}\;=\;\int_{0}^{L}\;B_{Q}{}^{T}\left(x\right)\;f_{t}\left(x\right)\;B_{Q}\left(x\right)\;dx$$

and s was the element residual deformation vector, Equation (7):

(7) $$s\;=\;\int_{0}^{L}\;B_{Q}{}^{T}\left(x\right)\;r\left(x\right)\;dx$$

The selection of the interpolation functions $B_{Q}\left(x\right)$ was derived from the assumption of constant axial force and linear bending distributions within the element. Finally, the determination of the element equilibrium equation could be accomplished by inversion of the element flexibility equation

(8) $${\left[F_{t}\right]}^{-1}\;(\Delta q-s)\;=\;\Delta Q$$

The element stiffness matrix $K_{t}$ could be obtained as $K_{t}=F_{t}{}^{-1}$. The element state determination was satisfied by the conversion of the element residual deformation s which could not be applied at the element nodes because it violates node compatibility, thus, the element nodal end force was applied to the element to impose end deformation − s using the current tangent element stiffness matrix. The force changed the element force field and yielded new section deformations that caused the new section residual r(x). For cases of current RC and R-SHCC columns, two iterations could offer sufficient convergence since the element residual deformation became sufficiently small. Instead of using displacement shape functions as the stiffness formulation, the current flexibility formulation applied force interpolation functions such as Equation (2) in order to satisfy equilibrium within an element. Therefore, the main advantage of the current flexibility formulation was that during the iterations the element force and deformation fields were adjusted until the section constitutive relations were satisfied, while always satisfying equilibrium along the element. In cases of nonlinear problems, therefore, this formulation could offer a more accurate solution not only in the element level but also in the structural level.

### 3.2. Isoparametric Finite Element with Layered Section Discretization

**Figure 8:**
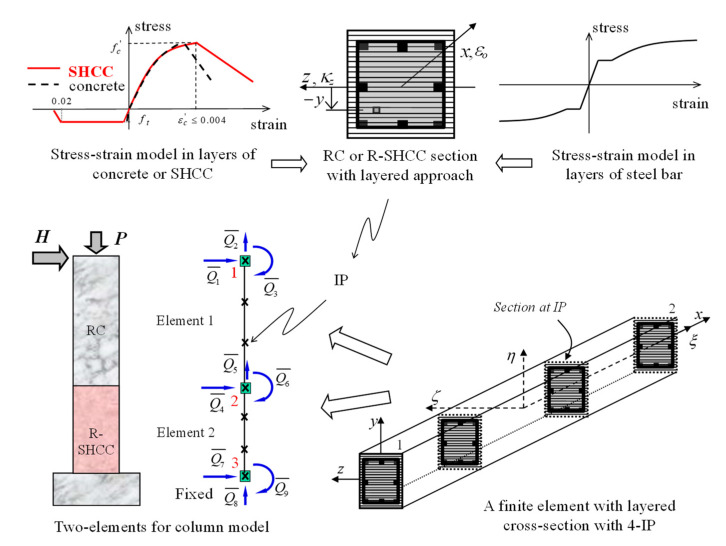
Isoparametric frame finite element and modeling of column specimens.

To model the beam-column finite element for RC and R-SHCC beam or column members, the element formulation relied on the layered cross-sectional discretization in order to compute the section force vector and section flexibility matrix, which were corresponding to the section deformation, as shown in Figure 8. The sectional force and deformation relation was established by the integration of the uniaxial stress–strain behavior of the layers.

As uniaxial laws, concrete and reinforcing steel bars were adopted as well-known stress and strain models [20,21], but SHCC after cracks had taken place in tension the tensile stress was sustained until the corresponding tensile strain reached to 2.0%, as shown in Figure 2c, where, $f_{T\;Y}$ was the tensile yielding stress of SHCC and $\varepsilon_{T\;Y}$ was the corresponding tensile yield strain of SHCC, and $f_{T\;U}\;$ was the tensile ultimate strength of SHCC and $\varepsilon_{T\;U}$ was the corresponding tensile ultimate strain of SHCC. Since section moment and section force were obtained by integrating all the layered stresses, moment and axial force were totally coupled. Therefore, the section tangential flexibility $f_{t}$ in Equations (3) and (6), by applying the integration in layered a cross-section, could be obtained from $f_{t}=k_{t}{}^{-1}$.

(9) $$k_{t}{}^{}\left(x\right)=\left[\begin{array}{cc} \sum_{ilyr=1}^{}E_{ilyr}^{}A_{ilyr}y_{ilyr}^{2} & -\sum_{ilyr=1}^{}E_{ilyr}^{}A_{ilyr}y_{ilyr}^{}\\ -\sum_{ilyr=1}^{}E_{ilyr}^{}A_{ilyr}y_{ilyr}^{} & \sum_{ilyr=1}^{}E_{ilyr}^{}A_{ilyr} \end{array}\right]$$

where, ilyr is the number of i-th layer in the cross-section, $E_{ilyr}\;$ is the tangential modulus in i-th layer of the cross-section, $A_{ilyr}$ is the cross-sectional area in i-th layer of the cross-section, and $y_{ilyr}$ is the distance from the middle of the cross-section to the i-th layer of the cross-section. The tangential modulus in each layer for SHCC, concrete, and reinforcing steel bar was updated according to the strain following the stress and strain curves for SHCC, concrete, or reinforcing steel bar, respectively, as shown in Figure 8.

Based on the description in this manuscript, a program of Matlab code was developed to analyze RC and R-SHCC beams, columns, and frames. Inelastic nonlinear finite element modeling and analysis of the RC and R-SHCC cantilever columns was also performed, corresponding with a series of experiments on three columns, as mentioned in the previous chapter. The cantilever column subjected to lateral load, H, combined with a constant axial load, P, was modeled as a distributed inelastic beam-column finite element formulation with a layered cross-sectional approach, as shown in Figure 8. Two specimens of RC and R-SHCC columns, RSH-s and RSH-n, were modeled as two finite elements as divided by two portions of concrete and SHCC, and each element had four integration points (IP). One specimen of a conventional RC column, RC-0, was modeled as both one and two finite elements to compare the accuracy of a nonlinear solution according to the number of elements. The integrals involved in the distributed inelastic beam-column element formulation were adopted numerically with the Gauss–Lobatto integration scheme because it always included the end cross-sections of the integration field in an element [19,22].

## 4. Comparison of Experiments and Finite Element Predictions of Columns

**Figure 9:**
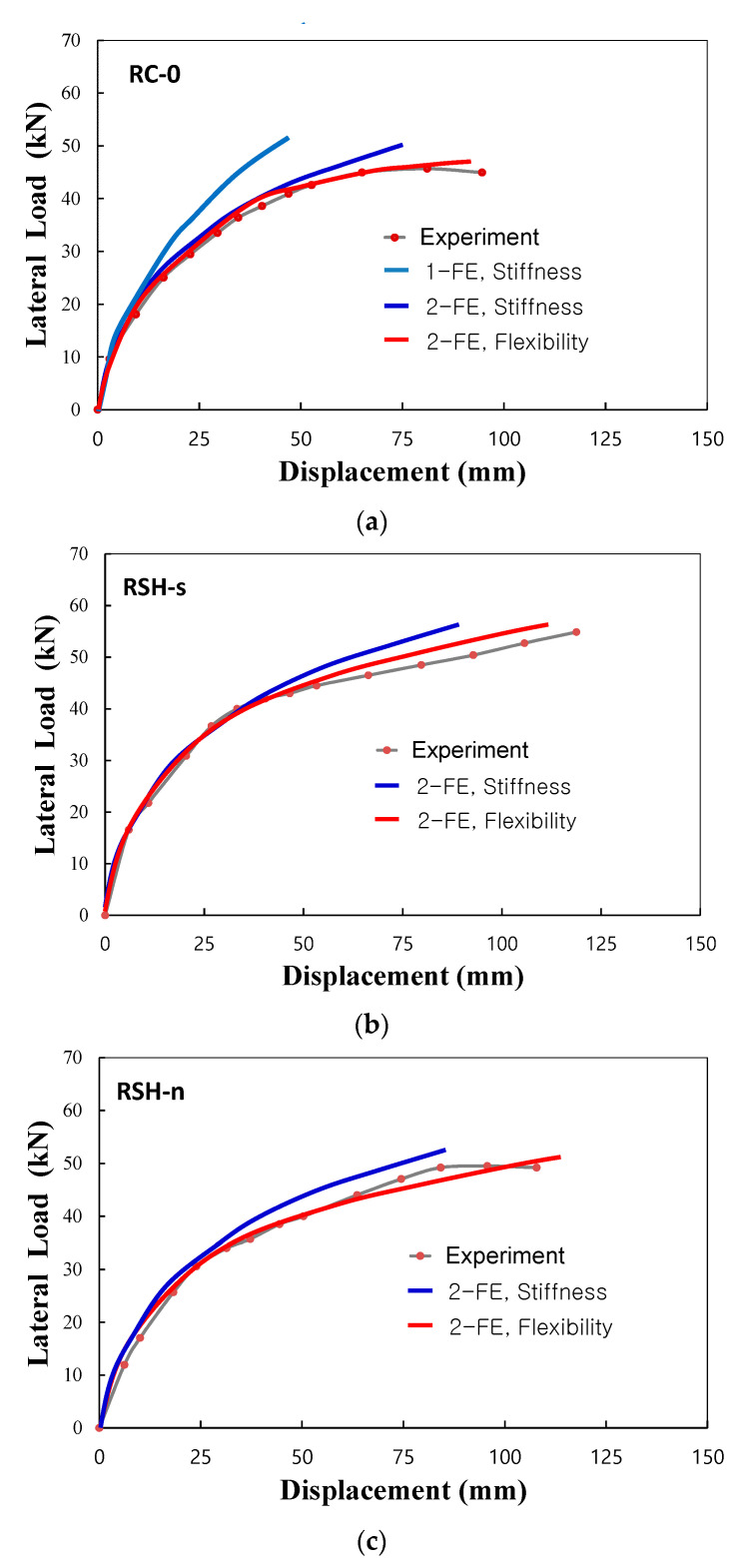
Experiments and predictions of lateral load–displacement responses, (**a**) RC-0, (**b**) RSH-s, (**c**) RSH-n.

From the experiments and the analysis of three column specimens, the lateral load and top displacement envelope curves of the columns are presented as shown in Figure 9. In the figure, 1-FE and 2-FE meant predicted curves obtained from analysis using one finite element and two finite elements, respectively, and Stiffness and Flexibility meant the predicted curves obtained from the stiffness finite element model and the flexibility finite model, respectively. For the RC column, RC-0, the maximum lateral load and top-displacement were measured as 45.7 kN and 94.6 mm, respectively, but the maximum responses of two R-SHCC columns exceeded the measurements of the RC column as recorded, 54.8 kN and 118.7 mm, respectively, for the specimen of RSH-s and 49.2 kN and 107.8 mm, respectively, for the specimen of RSH-n. In comparison of three specimens, two R-SHCC columns which were placed SHCC instead of concrete in the critical plastic hinge region showed enhanced lateral load and displacement capacities. As shown in Figure 9, the finite element predictions for load and displacement curves of three columns also manifested the same tendencies on the enhanced lateral load and displacement capacities of two R-SHCC columns.

**Figure 10:**
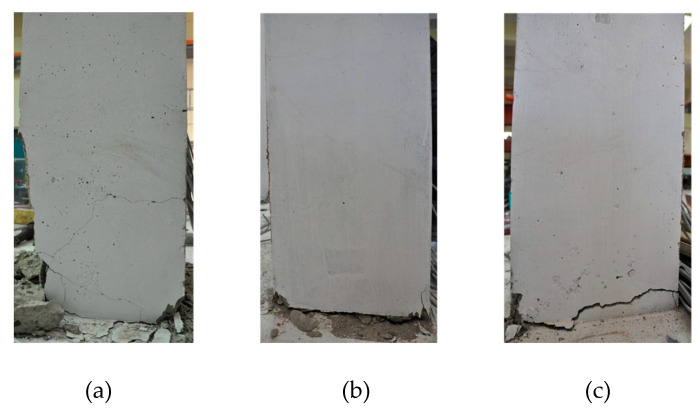
Damages and failure patterns of three column specimens: (**a**) RC-0; (**b**) RSH-s; (**c**) RSH-n.

The damages and failure patterns of three columns are shown in Figure 10. For the specimen of RC-0, the initial cracks were caused by bending taking place at a load of 16.8 kN near the column base, and the number of bending and shear cracks gradually increased according to the increase of the lateral load. After the yielding of the longitudinal tensile steel bars, the number of cracks was not so increased, but the opening of the cracks was rather prominent after the load exceeded about 38 kN. Finally, the RC column failed after reaching the maximum load, as observed from the buckling of the longitudinal steel bars, the spalling of cover concrete, the progress of shear cracks, the opening of widely bending cracks and damage to the concrete from the column base to the column plastic hinge region. For two specimens of RSH-s and RSH-n, after the initiations of cracks at loads of 16.2 kN and 15.5 kN, respectively, the number of multiple microcracks appeared according to the increase in the lateral load but were hard to recognize with one’s eyes. Even though tied reinforcing steel bars were not applied in the column plastic hinge regions, for the column of RSH-n, shear cracks were not observed in the region. Two R-SHCC columns finally reached bending failure by the excessive opening of the boundary interfaces between the column base and the footing without serious damages such as the spalling of cover concrete, buckling of reinforcing steel bars, and crushed damages of SHCC in compression, as shown in Figure 10.

The predicted lateral load and displacement curves by the two elements and beam-column finite element models were compared with experiments, as shown in Figure 9. For all cases of column specimens, the flexibility finite element model showed relatively accurate predictions but the stiffness finite element model showed relatively conservative results in predictions of nonlinear displacement responses. For the stiffness finite element models, the nonlinear displacement predicted by the one-element model was more greatly underestimated than by the two-element model, as shown in the case of RC column specimen RC-0. However, the flexibility finite element model showed comparatively accurate convergence in its estimations of nonlinear deformation responses regardless of the number of elements [19,22].

**Figure 11:**
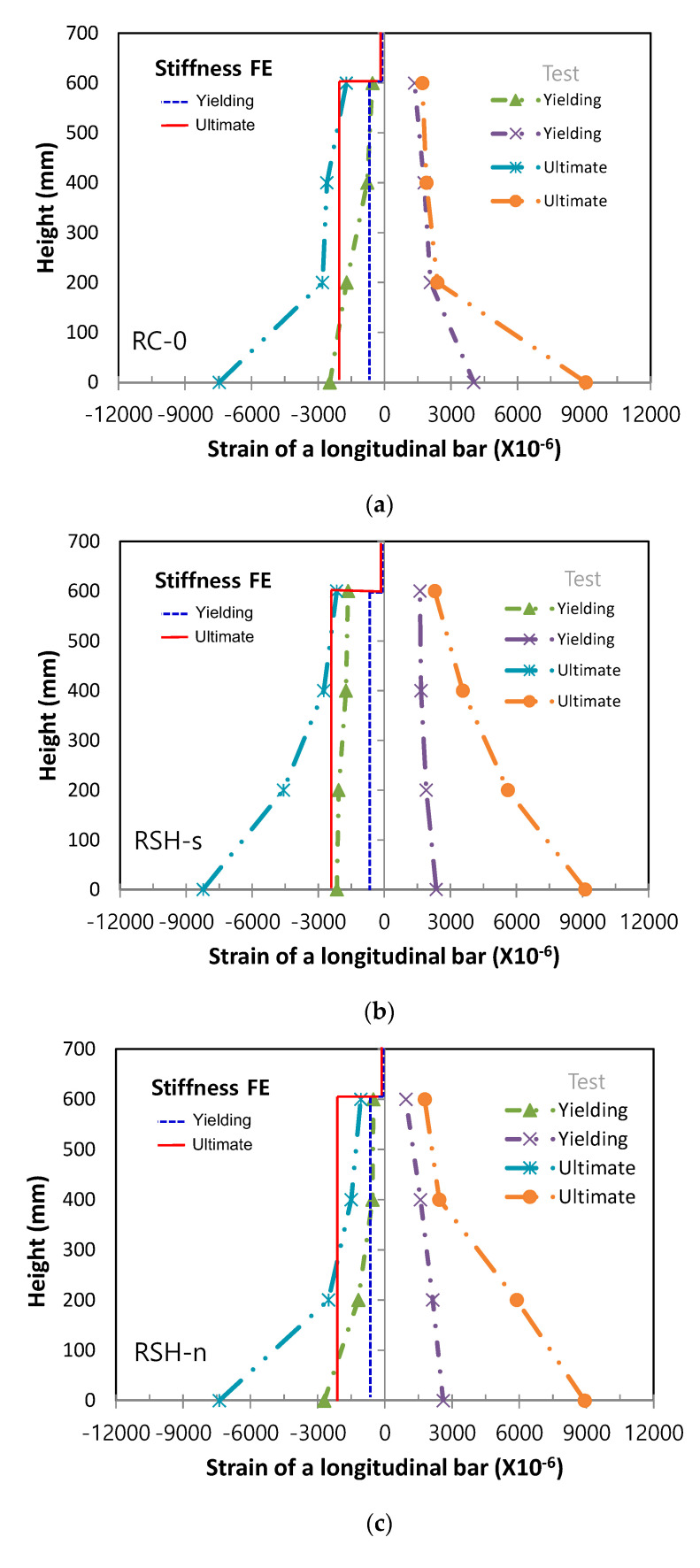
Experiments and stiffness finite element predictions for axial strains of steel bars, (**a**) RC-0, (**b**) RSH-s, (**c**) RSH-n.

**Figure 12:**
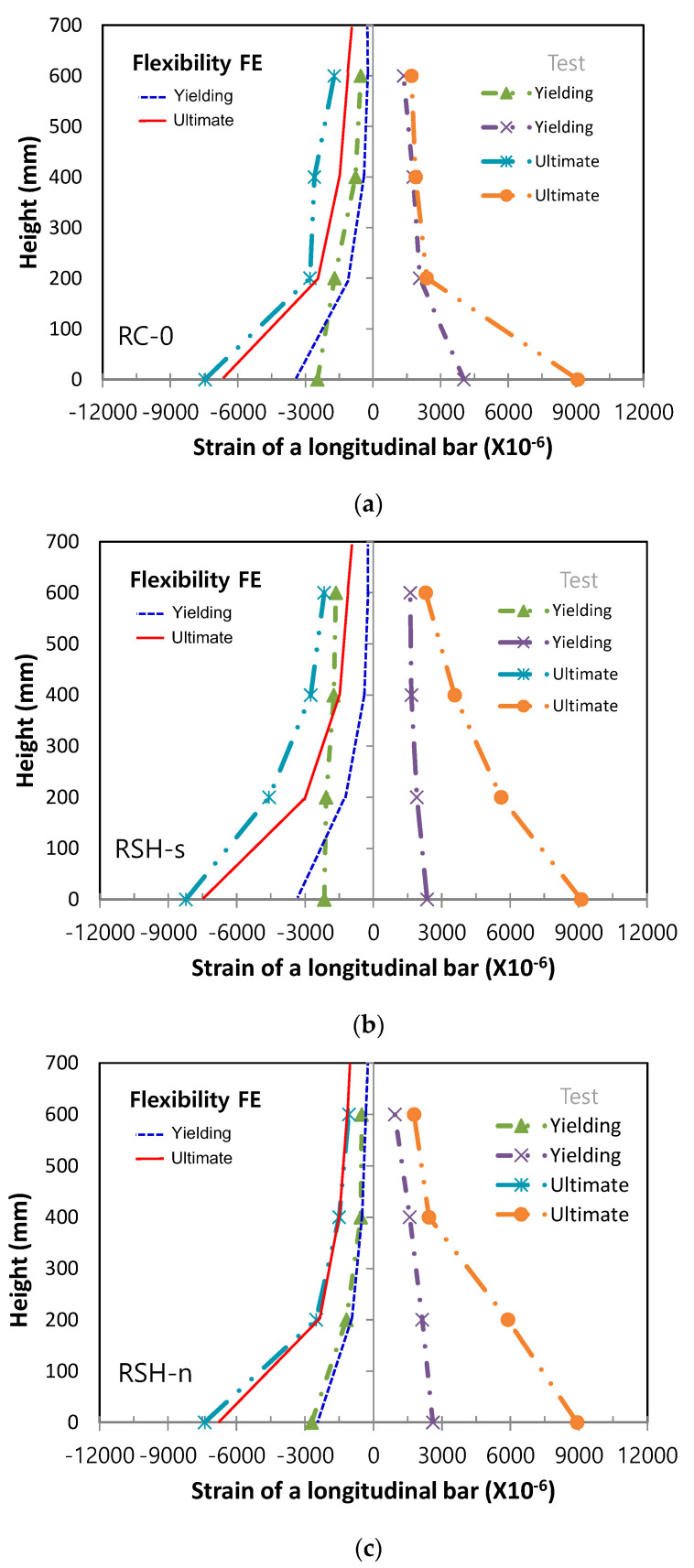
Experiments and flexibility finite element predictions for axial strains of steel bars, (**a**) RC-0, (**b**) RSH-s, (**c**) RSH-n.

For three column specimens, local nonlinear responses of cross-sections within the critical plastic hinge region were evaluated by measurements of axial strains in longitudinal steel bars, both at initial yielding of main steel bars and at the ultimate yielding, and the measured axial strains were plotted from the column base along the height of column, being compared with the predicted axial strains both by the stiffness model and flexibility model, respectively, as shown in Figure 11 and Figure 12. In two figures, Stiffness FE and Flexibility FE meant the predicted curves obtained from the stiffness finite element model and the flexibility finite model, respectively. For the column specimen of RSH-s, axial strains of the longitudinal bars at ultimate were distributed as smooth curves both in tensile and compressive steel bars. For the RC column specimen of RC-0, however, axial strains of the longitudinal bars at ultimate suddenly increased near the column base within the range of 200 mm. It could be explained that SHCC placed in the column plastic hinge region gave an effective advantage by preventing bending and shear cracks, spalling of the cover, and the buckling of steel bars, so as to minimize damage localizations in the region.

In comparison with the predicted axial strain distributions of longitudinal steel bars, the stiffness finite element method gave a constant distribution within an element along the height so that the predicted nonlinear responses in cross-sections didn’t provide accurate estimations. On the other hand, the flexibility finite element method gave a linear distribution of the axial strains between the integration points so that the predicted nonlinear responses in the cross-sections were able to be provided more accurately.

Laterally loaded columns, such as the columns in the current experiments, as well as lower story columns in buildings commonly reach failure by localized damages concentrated in the column plastic hinge zone. The accuracy of nonlinear problems in inelastic beam-column finite element models, therefore, mainly depends upon the estimation of nonlinear responses in local levels of cross-sections. The stiffness method could not satisfy equilibrium in an element, but the current flexibility method could satisfy equilibrium in an element because the element force and deformation fields were adjusted until the section’s constitutive relations were satisfied, while always satisfying equilibrium in an element. For the reason, the current flexibility method gave more accurate solution at both the element and structural levels, as manifested in the experiments and analysis solutions, as shown in Figure 10, Figure 11 and Figure 12.

## 5. Conclusions

In the current research, RC and R-SHCC columns subjected to lateral loads combined with a constant load were investigated by experiments and distributed inelastic beam-column finite element predictions, and the following conclusions were presented.

Instead of concrete, SHCC was applied in the column plastic hinge region as a scheme of minimizing damages in the region and the experiments showed that the use of SHCC could enhance the lateral load and displacement capacities of columns. Within the column plastic hinge region, SHCC could offer some effective advantages in the control of bending and shear cracks induced by multiple microcracks, the prevention of the spalling of cover, and the resistance of buckling of steel bars, so as to minimize damage localizations concentrated in the region.

Based on constitutive laws of SHCC considered with a proposed model of the post-cracked high-ductile tensile characteristic as well as concrete and reinforcing steel bars, the inelastic beam-column flexibility finite element model for the analysis of RC and R-SHCC columns was presented. In comparison with the well-known stiffness finite element method, the current flexibility method could give more accurate predictions of the lateral load and displacement responses of the column systems as well as of localized nonlinear responses of cross-sections as estimated in axial strains of longitudinal reinforcing steel bars.

## Figures and Tables

**Table 1:** Experimental variables of three column specimens.

Specimen Name	Types	Main Steel Bars	Tied Steel Bars
RC-0	RC	8-D13	D10@100
RSH-s	RC & R-SHCC	8-D13	D10@100
RSH-n	RC & R-SHCC	8-D13	No tied in SHCC

## Data Availability

Data sharing is not applicable to this article.
